# Saxagliptin for the treatment of type 2 diabetes mellitus: assessing cardiovascular data

**DOI:** 10.1186/1475-2840-11-6

**Published:** 2012-01-16

**Authors:** Michael E Cobble, Robert Frederich

**Affiliations:** 1 Canyons Medical Center, Sandy, UT, USA; 2 Bristol-Myers Squibb Company, Princeton, NJ, USA

**Keywords:** DPP-4 inhibitors, saxagliptin, type 2 diabetes mellitus, cardiovascular safety

## Abstract

Patients with type 2 diabetes mellitus (T2DM) are at high risk for cardiovascular (CV) disease; however, conclusive evidence that glycemic control leads to improved cardiovascular outcomes is lacking. Saxagliptin is a potent, selective dipeptidyl peptidase-4 inhibitor approved as an adjunct to diet and exercise to improve glycemic control in adults with T2DM. Saxagliptin was evaluated in a series of phase III trials as monotherapy; add-on therapy to metformin, a sulfonylurea, or a thiazolidinedione; and as initial therapy in combination with metformin. Saxagliptin consistently improved glycemic control (as reflected by significant decreases in glycated hemoglobin, fasting plasma glucose, and postprandial glucose compared with controls) and was generally well tolerated. In these analyses, saxagliptin had clinically neutral effects on body weight, blood pressure, lipid levels, and other markers of CV risk compared with controls. A retrospective meta-analysis of 8 phase II and phase III trials found no evidence that saxagliptin increases CV risk in patients with T2DM (Cox proportional hazard ratio, 0.43; 95% CI, 0.23-0.80 for major adverse cardiovascular events retrospectively adjudicated). Instead, it raised the hypothesis that saxagliptin may reduce the risk of major adverse CV events. A long-term CV outcome trial, Saxagliptin Assessment of Vascular Outcomes Recorded in Patients with Diabetes Mellitus-THrombolysis in Myocardial Infarction 53 (SAVOR-TIMI 53) is currently ongoing to determine whether saxagliptin reduces CV risk in T2DM.

## Introduction

It is well established that patients with type 2 diabetes mellitus (T2DM) are at increased risk of cardiovascular (CV) disease [1,2]. In addition to the chronic elevations in plasma glucose that contribute to increased CV risk [3,4], patients with T2DM often have comorbid conditions--such as obesity, hypertension, and dyslipidemia--that further contribute to the development of CV complications. As an example, the National Health and Nutrition Examination Survey (1999-2002) [5] revealed that patients with diabetes had mean body mass index (BMI) of 31.8 kg/m^2^, more than half reported having hypertension, and more than one third had dyslipidemia. Epidemiologic studies have shown a relationship between increasing levels of glycated hemoglobin (HbA_1c_) or fasting plasma glucose levels and the increased risk of CV complications, including coronary heart disease, chronic heart failure, and stroke; an association has also been shown between HbA_1c _levels and all-cause mortality [3,4].

Despite these epidemiologic findings, evidence for the benefit of improved glycemic control on CV events and mortality in patients with T2DM remains mixed. The 10 years of primary follow-up from the landmark UK Prospective Diabetes Study (UKPDS) [6] and 3 recent outcome studies (the Action to Control Cardiovascular Risk in Diabetes [ACCORD], Action in Diabetes and Vascular Disease: Preterax and Diamicron Modified Release Controlled Evaluation [ADVANCE] and Veterans Affairs Diabetes Trial [VADT]) all individually failed to demonstrate that intensive glycemic control reduces CV events and mortality. However, a subsequent meta-analysis increased the statistical power of these studies by combining them with the results of the PROactive trial [7] and was able to show that intensive glycemic control significantly reduces coronary events compared with standard glycemic control, without an increased risk of death [8]. Moreover, an additional 10-year follow-up from the UKPDS demonstrated a benefit of intensive glycemic control on the risk of myocardial infarction and all-cause mortality, but not on stroke or peripheral vascular disease [9].

In addition to conflicting data regarding the impact of intensive glycemic control on CV disease risk among patients with T2DM, the CV safety of the thiazolidinediones (particularly rosiglitazone) has come into question, ultimately leading the US Food and Drug Administration (FDA) to place severe restrictions on its use. The increased scrutiny also led the FDA to issue guidance recommendations in December 2008, requiring that all investigational antidiabetic agents demonstrate that treatment will not result in an unacceptable increase in CV risk, via meta-analysis of phase II and III trial data and/or large, long-term CV safety studies [10]. Although agents approved before these recommendations were not subject to this requirement, the CV safety of recently approved therapies (even those with studies designed before the 2008 guidance) has been carefully reviewed using the overall relative risk criteria defined by the FDA.

The current article reviews the CV safety of the selective dipeptidyl peptidase-4 (DPP-4) inhibitor saxagliptin, beginning with a brief overview of the rationale for use of this class of agents in T2DM. Phase III clinical trial data regarding CV risk factors are discussed, followed by the results of a meta-analysis of pooled data from phase II and phase III trials, conducted in accordance with the FDA guidance. The results of similar studies with other currently available DPP-4 inhibitors are summarized, as are the ongoing clinical trials to determine the impact of treatment with this class of agents on CV outcomes in patients with T2DM.

### Dipeptidyl Peptidase-4 Inhibitors: Rationale for Use

Dipeptidyl peptidase-4 is the enzyme that rapidly deactivates glucagon-like peptide-1 (GLP-1) and glucose-dependent insulinotropic polypeptide [11,12]; these incretin hormones, secreted from the gut in response to food intake, decrease postprandial glucose levels by stimulating insulin secretion, inhibiting glucagon secretion, and at pharmacologic concentrations, delaying gastric emptying [11,13]. Because the glycemic effects of DPP-4 inhibitors are glucose dependent and decline as postprandial serum glucose levels return to normal ranges, they are less likely to cause hypoglycemia. Consistent with this idea, low frequencies of hypoglycemic events have been observed during clinical use of DPP-4 inhibitors [14-19]. Moreover, these orally administered agents have demonstrated beneficial effects on pancreatic β-cell function [20-22].

Compared with other antihyperglycemic agents, DPP-4 inhibitors are associated with lower risks of hypoglycemia and weight gain than the sulfonylureas (SUs), lower risk of edema and chronic heart failure than the thiazolidinedione (TZDs), lower risk of diarrhea or gastrointestinal intolerance than metformin, and associated with fewer gastrointestinal adverse events than GLP-1 agonists [14-19]. Finally, as discussed in the next section, treatment with DPP-4 inhibitors has not been associated with increased CV risk [23-25]. The most frequently reported adverse reactions associated with DPP-4 inhibitors include headache, nasopharyngitis, and urinary tract and upper respiratory infections [26]. There have been spontaneous reports of pancreatitis among patients receiving DPP-4 inhibitors, including saxagliptin; however, there have been no increases in the incidence of pancreatitis events among DPP-4 inhibitors versus comparators in the pooled clinical experience of either saxagliptin, sitagliptin, [27] or vildagliptin [28]. Furthermore, 2 epidemiologic assessments of sitagliptin have failed to show an increase in pancreatitis events [29,30]. A clinical and scientific review of the evidence on DPP-4 inhibitors and pancreatitis calls for additional data [31]. In this setting of uncertainty, the US saxagliptin label calls for discontinuation of treatment for events of pancreatitis.

### Effects of Saxagliptin on Markers of Cardiovascular Risk

The core saxagliptin phase III trial program consisted of 6 multicenter, randomized, double-blind, 24-week studies that assessed the efficacy and safety of saxagliptin 2.5, 5, and 10 mg versus placebo as monotherapy in drug-naive patients [17,32]; saxagliptin 2.5, 5, and 10 mg versus placebo as add-on to metformin [18], saxagliptin 2.5 and 5 mg versus placebo as add-on to a TZD [15], or an SU (glyburide) [14]; and saxagliptin 5 and 10 mg as initial combination therapy with metformin versus metformin monotherapy in drug-naive patients [19]. In all of these studies, saxagliptin consistently improved glycemic control in patients with T2DM, as reflected by significant reductions in HbA_1c_, fasting plasma glucose, and postprandial glucose, as well as by the greater percentages of patients achieving the American Diabetes Association-recommended target HbA_1c _(< 7%) compared with controls. In addition, saxagliptin was generally well tolerated, with a low risk of hypoglycemia or weight gain. The 10-mg dose included in the phase III trials offered no efficacy benefit over the 5 mg dose and is therefore not an FDA-approved dose, despite an acceptable safety profile and no evidence of dose-limiting toxicity.

**Figure 1:**
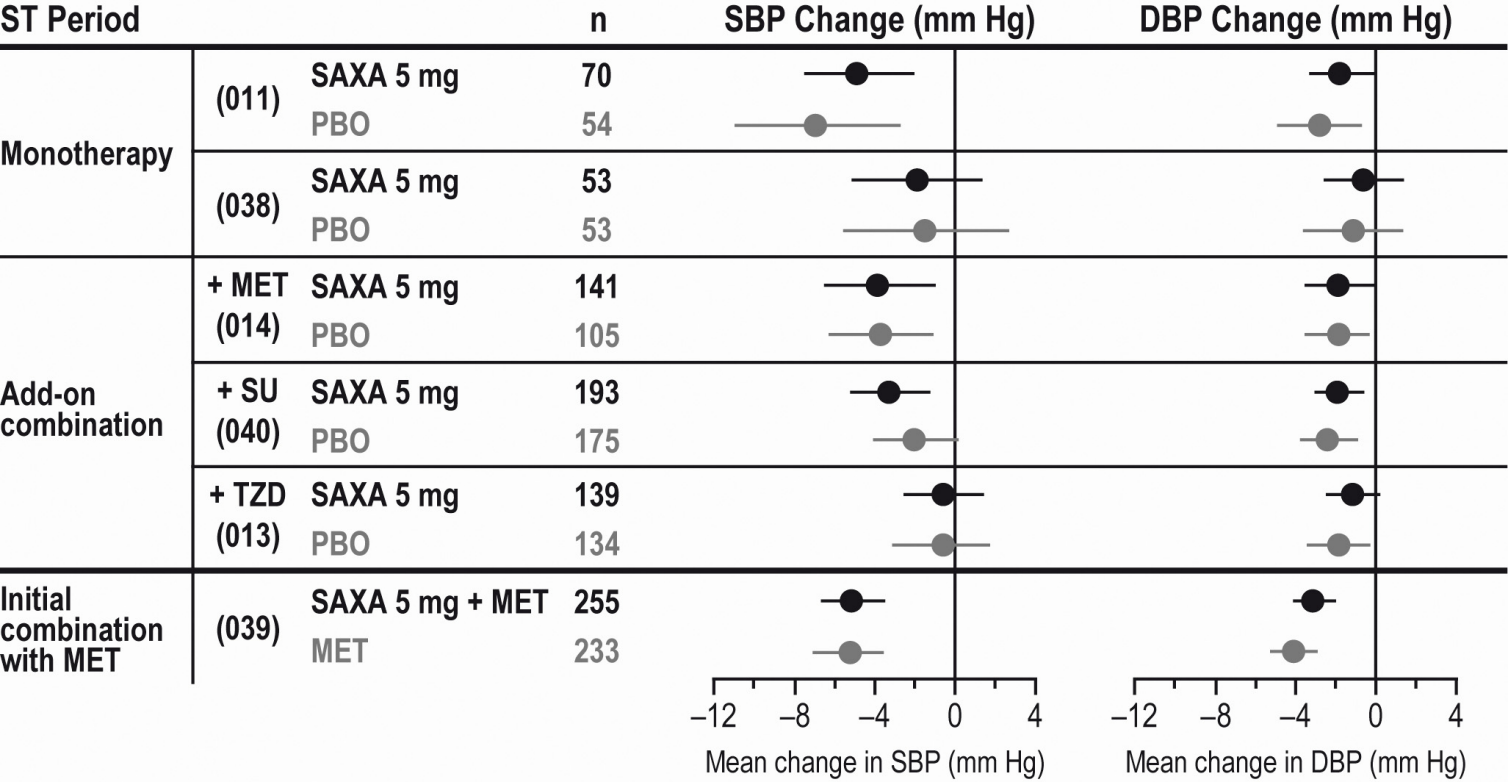
**Mean changes from baseline to 24 weeks in systolic and diastolic blood pressure **[23]. Forest plot shows the point estimate and 95% CI in the SAXA 5-mg and control groups. DBP = diastolic blood pressure; MET = metformin; PBO = placebo; SAXA = saxagliptin; SBP = systolic blood pressure; ST = short term; SU = sulfonylurea; TZD = thiazolidinedione.

**Figure 2:**
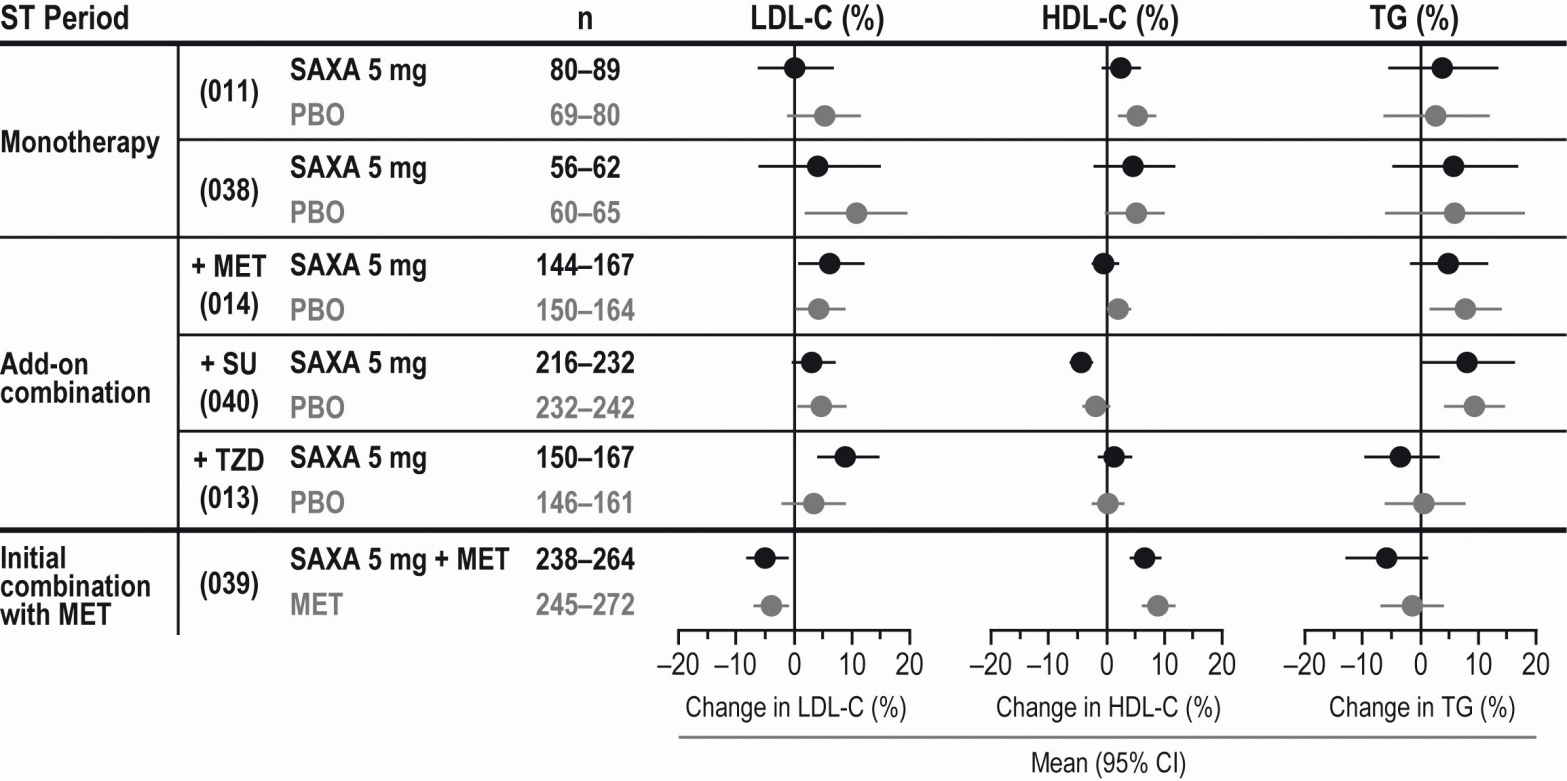
**Mean change from baseline to 24 weeks in LDL cholesterol, HDL cholesterol, and triglycerides **[23]. Forest plot shows the point estimate and 95% CI in the SAXA 5-mg and control groups. Measurements of each lipid parameter were not available for all patients; therefore, the number of patients (n) is presented as a range. HDL-C = high-density lipoprotein cholesterol; LDL-C = low-density lipoprotein cholesterol; MET = metformin; PBO = placebo; SAXA = saxagliptin; ST = short term; SU = sulfonylurea; TG = triglycerides; TZD = thiazolidinedione.

**Figure 3:**
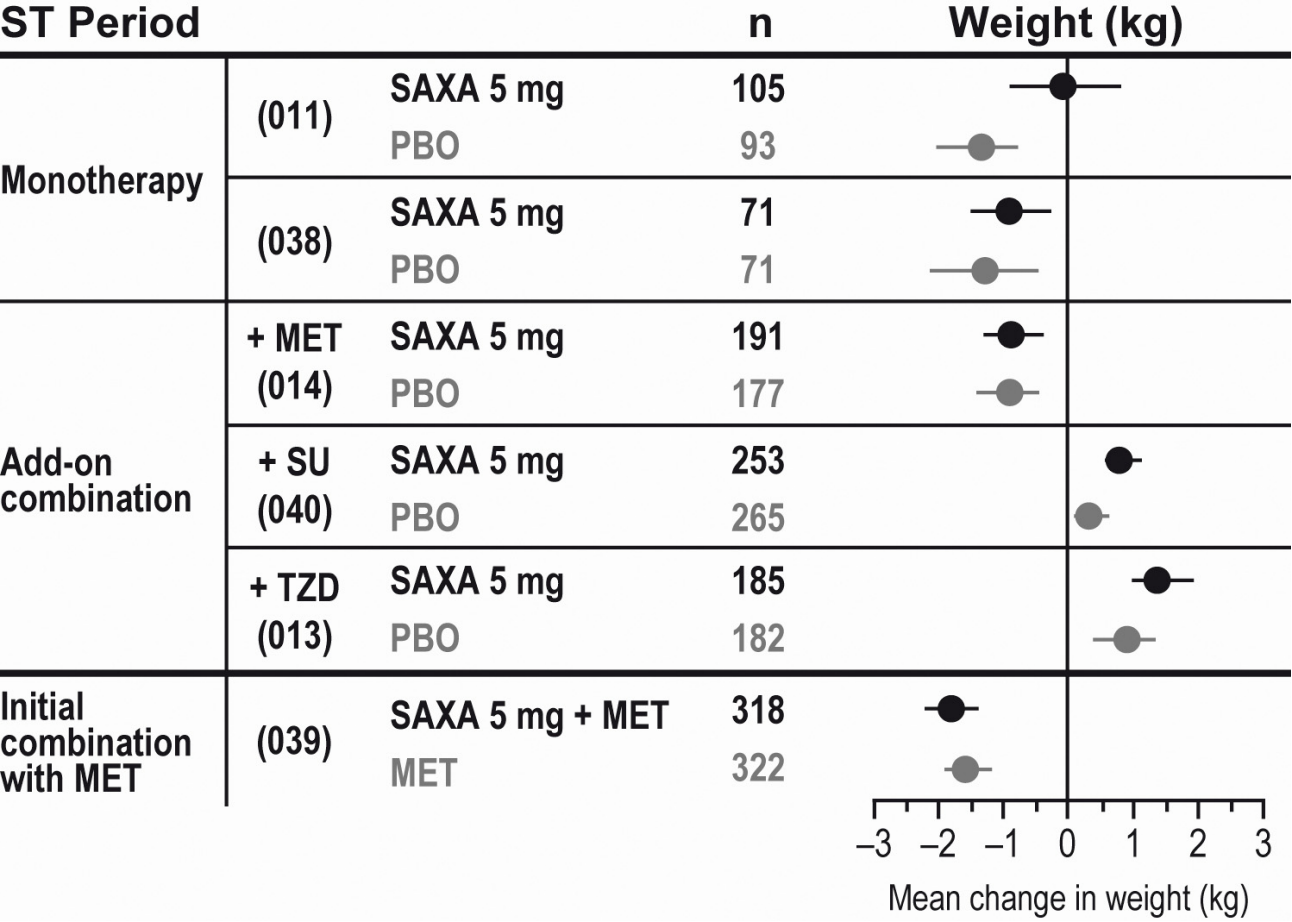
**Mean change from baseline to 24 weeks in body weight **[23]. Forest plot showing point estimate and 95% CI of the in the SAXA 5-mg and control groups. MET = metformin; PBO = placebo; SAXA = saxagliptin; ST = short term; TZD = thiazolidinedione; SU = sulfonylurea.

A pooled analysis of data from the phase III trials was conducted to assess the effects of saxagliptin on markers of CV risk. Blood pressure (multiple seated measurements), body weight, and lipid levels were routinely measured at multiple study visits. Descriptive statistics were summarized using observed values for blood pressure and last observation carried forward (LOCF) methods for lipid levels and body weight. Forest plots showing the point estimates and 95% CIs for the changes in these parameters with saxagliptin 5 mg and corresponding comparator groups are presented in Figures 1, 2 &3.

In all 6 pivotal phase III trials, blood pressure was reduced in the saxagliptin and control groups, and there were no clinically meaningful differences across groups (Figure 1). Changes in lipid parameters in the saxagliptin treatment groups generally paralleled the changes observed in the corresponding control groups (Figure 2). Low-density lipoprotein (LDL) showed small increases from baseline with saxagliptin 5 mg (and with placebo) in 4 clinical trials, no change in 1 trial, and a small decrease from baseline in 1 trial [33]. Small increases from baseline in high-density lipoprotein (HDL) were seen with saxagliptin monotherapy and with initial combination therapy with saxagliptin plus metformin, as well as in the corresponding control groups. Across the 3 studies of saxagliptin as add-on therapy, changes in HDL varied. Mean changes in triglycerides varied across all 6 studies, but were generally comparable between treatment groups within each trial [33].

Saxagliptin exhibited weight-neutral effects across the phase III clinical trials (Figure 3). At the 5-mg dose, mean body weight declined slightly from baseline in 3 trials, was essentially unchanged in 2 trials, and increased slightly in the trial of add-on to TZD (which may reflect, at least in part, the weight effects of the TZD). Similar small changes in body weight were seen with saxagliptin 2.5 mg (data not shown) [33].

### The Current Regulatory Environment for the Approval of Antidiabetic Agents

#### US Food and Drug Administration Guidance for Cardiovascular Safety

In December 2008, the FDA issued recommendations to provide meaningful data for estimating the CV risk associated with newer antidiabetic drugs [10]. This guidance recognizes that although improved long-term glycemic control measured by HbA_1c _leads to reduced risk of microvascular complications and remains an acceptable primary efficacy endpoint, T2DM is also associated with increased risk of CV disease--the primary cause of morbidity and mortality in T2DM patients.

To establish the safety of investigational antidiabetic medications, the FDA guidance calls for a systematic analysis (incorporating blinded adjudication) of CV death, myocardial infarction, and stroke using pooled data from phase II and phase III clinical trials, which should be designed to facilitate meta-analysis [10]. The relative risk ratio and its 2-sided 95% CI should be generated by comparing the number of CV events occurring in patients treated with the new drug versus those receiving control treatments. For an investigational antidiabetic drug to be considered for FDA approval, the results of this analysis must demonstrate that the upper bound of the 95% CI is < 1.8. If the overall risk-benefit analysis supports approval and the upper bound of the 95% CI is in the range of 1.3 to 1.8, a postmarketing CV safety trial will be necessary to document that the upper bound of the 95% CI is < 1.3. If the data available at the time of the new drug application show that the upper bound is < 1.3, a postmarketing CV safety trial may not be necessary [10].

#### Saxagliptin Meta-analysis

Although the saxagliptin trials intended for registration completed their primary analysis point before the 2008 FDA guidance, an assessment of investigator-identified CV events was completed in a meta-analysis of pooled data from 8 phase II and phase III clinical trials [23]. These trials involved a total of 3356 patients who received saxagliptin at doses ranging from 2.5 mg to 100 mg and 1251 patients who received a control treatment (placebo, metformin, uptitrated glyburide, or a TZD).

#### Methods

Cardiovascular events (death, myocardial infarction, stroke, and cardiac ischemic events) reported by investigators were systematically identified using a list of Medical Dictionary for Regulatory Activities (MedDRA) preferred-term diagnoses; all identified potential CV events subsequently went through independent adjudication by 2 independent reviewers. Patients in the saxagliptin groups were compared with those in the comparator groups; full CV event identification and statistical methods for this analysis have previously been published [23].

To obtain exposure-adjusted incidence rates for CV events in the meta-analysis of pooled data (and in accordance with the FDA guidance), the number of patients in each treatment group was divided by the number of patient-years of exposure, excluding exposure after the first event. The rates were presented per 1000 patient-years to adjust for exposure imbalances across treatment groups.

## Results

Within each trial and across all trials, the saxagliptin and comparator groups were generally balanced for baseline demographic and clinical characteristics, including median age (54 y vs 55 y), sex (51% vs 50% female), race (73% vs 71% white), mean BMI (30.4 kg/m^2 ^vs 30.3 kg/m^2^), mean HbA_1c _(8.5% vs 8.4%), and presence of at least 1 additional CV risk factor other than T2DM (81% vs 83%; including hypertension [52% vs 55%], hypercholesterolemia [44% vs 45%], and history of CV disease [12% vs 13%]).

The meta-analysis identified a total of 41 first major adverse CV events (MACE), including CV-related death, nonfatal myocardial infarction, and nonfatal stroke. These events occurred in 23 patients who received saxagliptin (0.7% of all saxagliptin-treated patients) and 18 patients who received comparators (1.4% of all comparator-treated patients) (Table 1). In addition, an independent clinical events committee performed a blinded, post-hoc, retrospective adjudication of all deaths and all events possibly representing a myocardial infarction and/or stroke from among all events coded to any of the 148 preferred terms representing possible ischemic events from 2 MedDRA standard queries: "myocardial infarction" and "central nervous system hemorrhages and cerebrovascular accidents" [23]. The results were close to the numbers of investigator-reported MACE (Table 1). From a total of 147 cases reviewed for potential MACE, 40 patients had confirmed events, including 22 saxagliptin-treated patients and 18 comparator-treated patients. Investigator reports and the independent clinical events committee identified 38 common patients with MACE; 3 patients were unique to the investigator report and 2 patients were unique to the adjudicated cases.

**Table 1:** Retrospective Analysis of CV Events With Saxagliptin Versus Comparators in Phase II/III Clinical Trials [23]

	Number of Patients (%)					
		
	SAXA 2.5 mg (n = 937)	SAXA 5 mg (n = 1269)	SAXA 10 mg (n = 1000)	All SAXA* (n = 3356)	Controls (n = 1251)	Hazard Ratio (95% CI)^†^
Investigator-reported MACE	6 (0.6)	6 (0.5)	11 (1.1)	23 (0.7)	18 (1.4)	0.44 (0.2-0.82)
Adjudicated MACE	6 (0.6)	7 (0.6)	9 (0.9)	22 (0.7)	18 (1.4)	0.42 (0.23-0.80)
Myocardial infarction^**‡**^ Stroke^**‡**^ Other CV deaths^**§**^	2 (0.2) 4 (0.4) 0 (0)	4 (0.3) 4 (0.3) 0 (0)	2 (0.2) 3 (0.3) 4 (0.4)	8 (0.2) 11 (0.3) 4 (0.1)	8 (0.6) 5 (0.4) 6 (0.5)	
All deaths	3 (0.3)	3 (0.2)	4 (0.4)	10 (0.3)	12 (1.0)	
CV deaths^||^	1 (0.1)	2 (0.2)	4 (0.4)	7 (0.2)	10 (0.8)	

CV = cardiovascular; MACE = major adverse cardiovascular event; SAXA = saxagliptin.

*Includes 150 patients who received saxagliptin 20, 40, and 100 mg daily in a dose-ranging trial.

^†^Cox proportional hazard ratio (95% CI) for all saxagliptin vs controls.

^‡^Includes patients with fatal and nonfatal events; patients with a myocardial infarction and stroke were counted in each category.

^§^CV deaths that could not be clearly determined as myocardial infarction or stroke.

**^||^**Investigator-reported and committee-adjudicated CV death rates were identical.

Table reprinted from Postgraduate Medicine, (3)122, Frederich R, Alexander JH, Fiedorek FT et al. A Systematic Assessment of Cardiovascular Outcomes in the Saxagliptin Drug Development Program for Type 2 Diabetes, page 20. Copyright 2010, with permission from JTE Multimedia.

In the analysis of relative risk with saxagliptin, the Cox proportional hazard ratio was 0.44 (95% CI, 0.24-0.82) for investigator-based assessments and 0.43 (95% CI, 0.23-0.80) for independently adjudicated events [23]. The numbers of patients with a CV event per 1000 patient-years of follow-up are lower with all saxagliptin regimens combined than with all comparators (Table 2). This finding suggested that saxagliptin may reduce CV risk in patients with T2DM, a hypothesis that will be evaluated in the Saxagliptin Assessment of Vascular Outcomes Recorded in Patients with Diabetes Mellitus-Thrombolysis in Myocardial Infarction 53 (SAVOR-TIMI 53) trial [34].

**Table 2:** CV Event Rate (Incidence [SD]) per 1000 Patient-Years [47]

	All Saxagliptin* (n = 3356)	Controls (n = 1251)
Standard MedDRA query MACE^†^	28.4 (2.9)	31.9 (5.0)
Acute CV events	10.7 (1.8)	17.6 (3.7)
Custom MACE^‡^	6.2 (1.3)	13.1 (3.2)
Primary MACE^§^	6.2 (1.3)	13.9 (3.3)

CV = cardiovascular; MACE = major adverse cardiovascular event; MedDRA = Medical Dictionary for Regulatory Activities.

*Includes 150 patients who received saxagliptin 20, 40, and 100 mg daily in a dose-ranging trial. ^†^Standard MedDRA query MACE is a composite endpoint of CV death and all standard MedDRA query preferred terms for "myocardial infarction" and "central nervous system hemorrhages and cerebrovascular accidents."

^‡^Custom MACE is a composite endpoint of CV death, MI and stroke using MedDRA preferred terms indicative of MACE suggested by the FDA.

^§^Primary MACE includes adverse events of myocardial infarction, stroke, or CV death developed by the sponsor. In practice, custom MACE differed from primary MACE only by a single subject which the investigator updated to an event of anterior MI. Such an update was allowed by the sponsor rules.

### Cardiovascular Risk With Other Dipeptidyl Peptidase-4 Inhibitors

Pooled analyses of nonadjudicated events with sitagliptin and adjudicated CV-related events with vildagliptin, linagliptin, and alogliptin have been reported. The relative risk for sitagliptin versus comparators was 0.68 (95% CI, 0.41-1.12) [24]. With vildagliptin, relative risks of cardiocerebrovascular events were 0.88 (95% CI, 0.37-2.11) with vildagliptin 50 mg once daily and 0.84 (95% CI, 0.62-1.14) with vildagliptin 50 mg twice daily [25]. Data from a prespecified, prospective meta-analysis of the linagliptin phase III studies showed a relative risk of CV-related events with linagliptin versus comparators of 0.34 (95% CI, 0.16-0.70) [35]. Meta-analysis of phase II and III studies with alogliptin versus placebo calculated a relative risk of 0.63 (95% CI, 0.21-1.91) [36].

### Ongoing Cardiovascular Outcome Studies

Saxagliptin is being evaluated in the SAVOR-TIMI 53 trial (ClinicalTrials.gov identifier NCT01107886) [34], to demonstrate CV safety. Additionally, based on the results of the saxagliptin meta-analysis, the SAVOR-TIMI 53 trial was also powered to determine whether saxagliptin can reduce the risk of CV events. The study plans to enroll 16,500 patients with T2DM who have HbA_1c _≥6.5% and a high risk for CV events (defined as established CV disease and/or multiple risk factors). Eligible patients will be randomly allocated to receive saxagliptin (5 mg for patients with normal or mildly impaired renal function or 2.5 mg for those with moderate renal impairment) or placebo once daily during the approximately 5-year study period, the projected timeframe necessary to observe 1,040 MACE. The primary outcome is a composite of CV death, nonfatal myocardial infarction, or nonfatal ischemic stroke; completion is expected by the middle of 2015.

Sitagliptin is being evaluated in the TECOS trial (Randomized, Placebo-Controlled Clinical Trial to Evaluate Cardiovascular Outcomes After Treatment With Sitagliptin in Patients With T2DM and Inadequate Glycemic Control trial [NCT00790205]) [37], which is designed to assess CV outcomes with sitagliptin (100 mg once daily; or 50 mg for those with moderate renal impairment) versus placebo in patients older than 50 years with preexisting CV disease and HbA_1c _of 6.5% to 8.0% receiving usual care including other antihyperglycemic agents. The primary outcome measure is time to first CV event (a composite of CV death, nonfatal myocardial infarction, nonfatal stroke, or unstable angina requiring hospitalization). The study plans to enroll 14,000 patients who will be treated and followed up for ≤5 years, until 1300 CV events are observed; study completion is expected in December 2014 [38].

Linagliptin, the newest DPP-4 inhibitor (approved by the FDA in May 2011), will be evaluated for CV outcomes in the CAROLINA trial (Multicentre, International, Randomised, Parallel-Group, Double-Blind Study to Evaluate Cardiovascular Safety of Linagliptin Versus Glimepiride in Patients With Type 2 Diabetes Mellitus at High Cardiovascular Risk [NCT01243424]) [39]. The planned study population is 6000 patients with T2DM who have preexisting or T2DM-related CV disease, are older than 70 years, or have ≥2 specified CV risk factors. The primary endpoint is time to first occurrence of CV death, nonfatal myocardial infarction, nonfatal stroke, or hospitalization for unstable angina pectoris; study completion is expected in 2018.

The effect of alogliptin on CV outcomes is being investigated in patients with T2DM who have acute coronary syndrome in the EXAMINE trial (Examination of Cardiovascular Outcomes: Alogliptin vs Standard-of-Care in Patients With T2DM and Acute Coronary Syndrome trial [NCT00968708]) [40]. This study plans to enroll 5400 patients (HbA_1c _6.5%-11.0% on monotherapy or combination antidiabetic therapy, or 7.0%-9.0% if the regimen includes insulin) who have a diagnosis of acute coronary syndrome within 15 to 90 days before randomization. Patients will be randomly allocated to receive alogliptin or placebo once daily for up to 4.75 years, each in addition to the standard of care; the daily dose of alogliptin will be 25 mg for patients with normal or mildly impaired renal function, 12.5 mg for those with moderate renal impairment, and 6.25 mg for those with severe renal impairment or end-stage renal disease. The primary outcome is a composite of CV death, nonfatal myocardial infarction, nonfatal stroke, and urgent revascularization due to unstable angina, with study completion expected in 2014.

## Conclusion

Recent concerns regarding CV safety have been raised by data from various studies of traditional diabetes treatment regimens [41], rosiglitazone [42], and SUs [43,44]; thus, attention has focused on the CV safety of all antidiabetic medications. Six similarly designed 24-week trials of the DPP-4 inhibitor saxagliptin as monotherapy, add-on therapy, or initial combination therapy with metformin showed that saxagliptin does not adversely affect blood pressure, lipid levels, body weight, or other CV markers compared with control treatments.

In accordance with the FDA guidance for establishing the CV safety of new antidiabetic drugs, a retrospective meta-analysis was conducted using pooled data from the saxagliptin phase II and phase III clinical trials. The meta-analysis found no evidence that saxagliptin increases CV risk in patients with T2DM, and a long-term outcome study (SAVOR-TIMI 53) is currently ongoing to test the hypothesis that saxagliptin treatment may even reduce CV risk [34]. Meta-analyses of other DPP-4 inhibitors [37,39,40,45,46] have also failed to detect an increased CV risk associated with this drug class; outcome studies in patients at high risk for CV events are also underway for sitagliptin, alogliptin, and linagliptin [37,39,40,45,46]. Thus, a substantial body of data supporting the CV safety of DPP-4 inhibitors has already been generated, and in the next few years, an unprecedented amount of clinical outcome data for saxagliptin and other DPP-4 inhibitors will address the concerns and possible benefits of DPP-4 inhibitors in diabetes-associated CV disease.

In conclusion, the incretin medication class is currently approved as monotherapy or combination therapy in adults with T2DM and has been shown to be beneficial in glucose management and the achievement of HbA_1c _goals. Although early analyses on CV safety and possible benefits are promising, definitive data have not yet been generated. Future and ongoing CV morbidity and mortality studies across the DPP-4 inhibitor drug class will offer more insight into the risks and rewards of diabetes care and its associated outcomes.

## Abbreviations

BMI: body mass index; CV: cardiovascular; DPP-4: dipeptidyl peptidase-4; FDA: US Food and Drug Administration; GLP-1: glucagon-like peptide-1; HbA_1c_: glycated hemoglobin; HDL: high-density lipoprotein; LDL: low-density lipoprotein; LOCF: last observation carried forward; MACE: major adverse cardiovascular events; MedDRA: Medical Dictionary for Regulatory Activities; SAVOR-TIMI 53: Saxagliptin Assessment of Vascular Outcomes Recorded in Patients with Diabetes Mellitus-Thrombolysis in Myocardial Infarction 53; SU: sulfonylurea; T2DM: type 2 diabetes mellitus; TZD: thiazolidinedione; UKPDS: UK Prospective Diabetes Study.

## Competing interests

Michael Cobble reports that he has served on advisory boards for Abbott and Genentech; speaker boards for Abbott, AstraZeneca, Bristol-Myers Squibb Company, Eli Lilly, Forest Laboratories, and GlaxoSmithKline; has been involved in research for Johnson & Johnson; and served as Chief Medical Officer for Atherotech Diagnostics. Robert Frederich is an employee of Bristol-Myers Squibb and holds stock in the company.

## Authors' contributions

MC and RF participated in the collection and assessment of study data included in this manuscript. Both authors helped in the drafting and revisions of this manuscript and have approved the final submitted version.

## Author information

MC is an adjunct professor at the University of Utah School of Medicine and is the Clinical Practice Director of Canyons Medical Center in Sandy, UT. He is a board certified Clinical Lipidologist, Fellow of the National Lipid Association (FNLA), Certified Hypertension Specialist (ASH), and member of ADA, AHA, ACC, ASA, and AAFP. RF served as medical director/monitor on 3 of the saxagliptin phase 3 trials presented in his role at Bristol-Myers Squibb. He has previously held faculty positions at the University of Kentucky and at Harvard in the Endocrinology Division.
